# 

**Published:** 1876-07

**Authors:** 


Vol. VI. July, 1876. No. 7

S.G.O. INDEXED

### THE SOUTHERN MEDICAL RECORD:

PUBLISHED MONTHLY AT

ATLANTA, GEORGIA.

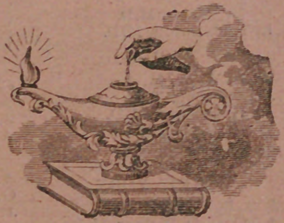

LOCAL EDITORS:

THOS. S. POWELL, M.D. R. C. WORD, M.D

W. T. GOLDSMITH, M.D. H. B. LEE, M.D.

ASSOCIATE EDITORS:

T. CURTIS SMITH, M.D. ..........Middleport, Ohio.
SWAN M. BURNETT, M.D .......Knoxville, Tenn.
ALBAN S. PAYNE, M.D..........Markham's, Va.
A. R. KILPATRICK, M.D..........Navasota, Texas.
A. A. LYON, M.D....................Shreveport, La.
G. A. BAXTER, M.D ..............Chattanooga, Tenn.
J. B. MATTISON, M.D.............Brooklyn, N. Y.
E. T. EASLY, A.M., M.D..........Little Rock, Ark.

*"QUICQUID PRÆCIPIES ESTO BREVIS."*

ATLANTA, GA.:
JAS. P. HARRISON & CO., PRINTERS AND BINDERS.
1876.

